# Managing Sleep in Bipolar Disorder: An Overview of Pharmacological and Non‐Pharmacological Interventions

**DOI:** 10.1111/bdi.70179

**Published:** 2026-09-02

**Authors:** Valentina Baldini, Francesco Pasquino, Francesca Iannucci, Noemi Venezia, Diana De Ronchi, Giuseppe Plazzi, Lorenzo Pelizza, Marco Menchetti

**Affiliations:** ^1^ Department of Biomedical and Neuromotor Sciences University of Bologna Bologna Italy; ^2^ Department of Biomedical, Metabolic and Neural Sciences University of Modena and Reggio Emilia Bologna Italy; ^3^ IRCCS Istituto Delle Scienze Neurologiche di Bologna Bologna Italy

**Keywords:** bipolar disorder, circadian rhythm, non‐pharmacological interventions, sleep disturbances

## Abstract

**Objectives:**

Sleep disturbances are common in individuals with bipolar disorder (BD) and are linked to greater symptom severity, higher relapse risk, and increased suicidality. Despite their clinical relevance, sleep problems in BD are frequently underdiagnosed and inadequately treated. This systematic review aimed to evaluate the efficacy and safety of pharmacological and non‐pharmacological interventions specifically targeting sleep disturbances in BD.

**Methods:**

We systematically searched PubMed, Embase, Web of Science, and PsycINFO for studies published until May 2025. Inclusion criteria encompassed randomized controlled trials and observational studies assessing interventions specifically targeting sleep disturbances in individuals with BD.

**Results:**

Eleven studies met the inclusion criteria. Non‐pharmacological interventions, particularly cognitive behavioral therapy for insomnia adapted for bipolar disorder (CBTI‐BD), consistently improved sleep efficiency, latency, and total sleep time and were associated with reductions in depressive symptoms. Bright light therapy yielded mixed results, whereas the Benson Relaxation Technique showed improvements in sleep quality and emotional regulation. Among pharmacological approaches, melatonin improved circadian alignment and sleep parameters without mood destabilization. Suvorexant showed limited efficacy. Antidepressants were linked to poorer sleep quality and greater impulsivity during euthymia.

**Conclusions:**

Sleep‐focused interventions, especially behavioral strategies such as CBTI‐BD, appear effective and well tolerated for managing sleep disturbances in BD, primarily in euthymic and interepisode samples where they have been tested. Pharmacological interventions show more variable effects and require careful attention to tolerability and clinical stability. Because each intervention was evaluated in a single clinical phase, the available evidence cannot determine whether clinical phase or chronotype moderates treatment response; trials designed to detect such moderation are needed before individualized, phase‐specific recommendations can be made.

## 1 Introduction

Sleep disturbances are among the most common and persistent features of bipolar disorder (BD), affecting individuals not only during acute mood episodes but also throughout euthymic phases [1]. Over their lifespan, individuals with BD experience a wide range of sleep alterations, including insomnia, hypersomnia, increased variability in sleep timing, decreased sleep efficiency, delayed sleep onset, and disruptions to their circadian rhythm [2, 3].

Longitudinal studies have consistently linked sleep disruption to a greater vulnerability to relapse, a poorer illness trajectory, impaired functioning, and a heightened risk of suicidal behavior [4, 5]. Furthermore, abnormalities in sleep architecture and rest–activity rhythms often persist even during periods of clinical stability, suggesting that sleep dysfunction may serve as a trait marker of BD rather than just an epiphenomenon of mood states [6, 7].

Early sleep disturbances are often detectable during the prodromal phase of mood episodes and even at the onset of illness, indicating their potential role in the pathogenesis of BD. Similar patterns are observed in other severe psychiatric disorders, such as psychosis, where sleep disruption typically precedes the emergence of clear psychopathology and may serve as an early clinical warning sign [8, 9, 10].

The pathophysiology of sleep dysregulation in BD involves a complex interplay of genetic, neurobiological, and psychosocial factors. Variants in circadian genes such as CLOCK, ARNTL2, and PER3 have been implicated in BD, along with evidence of altered melatonin secretion, delayed sleep–wake cycles, and a predominance of evening chronotypes in affected individuals [11]. Disruptions in social rhythms—such as irregular daily routines and interpersonal stress—may further destabilize circadian functioning and precipitate mood episodes, forming the theoretical basis for interventions like Interpersonal and Social Rhythm Therapy (IPSRT) [12]. Additionally, the bidirectional relationship between sleep and mood supports the hypothesis that targeting sleep regulation may contribute to mood stabilization and the prevention of mood recurrence [13]. Despite the high prevalence of sleep disturbances in BD, their clinical management remains often inadequate. Sedative‐hypnotic medications commonly used to treat primary insomnia, such as benzodiazepines and Z‐drugs, have been associated with potential risks of misuse and abuse, especially in individuals with BD, although true physiological dependence appears to be relatively rare [14].

Furthermore, these treatments may alleviate symptoms without addressing the underlying circadian dysregulation. Alternative pharmacological strategies, such as melatonin receptor agonists and orexin antagonists, have demonstrated promise in small trials and observational studies [15, 16]. At the same time, interest in non‐pharmacological interventions has increased. Cognitive‐behavioral approaches tailored for BD, such as CBT for Insomnia adapted for BD (CBTI‐BD), IPSRT, and relaxation‐based techniques, have shown improvements in sleep quality and, in some instances, mood stabilization and relapse prevention [17]. However, current studies differ significantly in design, participant characteristics, outcome measures, and follow‐up duration, making direct comparisons and generalization difficult.

This systematic review aims to synthesize current evidence from randomized controlled trials and observational studies on pharmacological and behavioral interventions for sleep disturbances in BD. Specifically, it examines (I) the types of interventions employed, (II) the sleep‐related outcomes assessed, (III) the impact of these interventions on mood stability and clinical course, and (IV) the incidence of adverse events. By integrating findings across therapeutic domains, this review seeks to inform clinical decision‐making and guide the development of integrated, phase‐specific treatment strategies for sleep disturbances in BD.

## 2 Methods

This systematic literature review was conducted and reported according to the Preferred Reporting Items for Systematic Reviews and Meta‐Analyses (PRISMA) guidelines [18]. The study protocol was registered in advance on PROSPERO (CRD420251024576).

### 2.1 Eligibility Criteria

To be included in the review, studies had to investigate sleep‐targeted interventions in individuals diagnosed with BD, either Type I or II, based on standardized diagnostic criteria such as the DSM or ICD. Eligible interventions encompassed both pharmacological and non‐pharmacological approaches to improve sleep parameters or regulate the circadian rhythm. Examples include melatonin, sedative‐hypnotics, CBTI‐BD, light therapy, and chronotherapy. Only studies that reported clinical outcomes such as mood stabilization, relapse prevention, improvement in sleep quality or architecture, regulation of circadian rhythms, suicidality, or general psychiatric functioning were considered. The review included randomized controlled trials, cohort studies, and case–control studies published in peer‐reviewed journals. Only articles written in English were considered.

Studies were excluded if they examined sleep disturbances in BD without evaluating the impact of a specific intervention or if they were case reports, narrative reviews, editorials, commentaries, or non‐peer‐reviewed articles. Retracted publications were also excluded.

### 2.2 Search Strategy

A systematic search was conducted on PubMed, Web of Science, EMBASE, and PsycINFO from January 2015 to May 2025 using the following search criteria (“bipolar disorder” OR “bipolar spectrum” OR “manic‐depressive disorder”) AND (“sleep” OR “sleep disorders” OR “insomnia” OR “hypersomnia” OR “circadian rhythm” OR “sleep quality” OR “sleep deprivation” OR “chronotherapy” OR “light therapy” OR “melatonin”) AND (“treatment” OR “therapy” OR “intervention” OR “cognitive behavioral therapy” OR “pharmacological intervention” OR “non‐pharmacological intervention” OR “mood stabilization” OR “relapse prevention”). This 10‐year timeframe was selected to ensure the inclusion of recent and clinically relevant evidence, reflecting current diagnostic frameworks, therapeutic approaches, and methodological standards in psychiatric research.

### 2.3 Selection of the Studies

All records identified through the database searches were imported into reference management software, and duplicates were removed. The selection process occurred in two phases. In the first phase, two independent reviewers screened titles and abstracts to assess their potential relevance based on the predefined eligibility criteria. In the second phase, full‐text versions of the potentially eligible articles were retrieved and examined in detail to determine final inclusion. Discrepancies between reviewers at any stage were resolved through discussion, and if consensus could not be reached, a third reviewer was consulted. In addition, the retraction status of all records considered for inclusion was verified against PubMed and the publishers' websites, and retracted articles were excluded. The study selection process will be documented and presented in a PRISMA flow diagram.

### 2.4 Data Extraction

Data were extracted using a standardized form developed specifically for this review. Two independent reviewers conducted the extraction, and any discrepancies were resolved through discussion or, when necessary, with the involvement of a third reviewer. Detailed information was collected from each included study regarding authorship, year of publication, country of origin, and study design. Special attention was given to identifying whether the study was a randomized controlled trial, a cohort study, or a cross‐sectional investigation.

Information about participants included the total number of individuals enrolled, the diagnostic criteria used to confirm bipolar disorder, the subtype of BD (Type I, Type II, or not otherwise specified), and the clinical state of participants at baseline, such as whether they were euthymic, experiencing a depressive episode, or in an acute affective state.

The characteristics of the interventions were meticulously recorded, including whether the approach was pharmacological or non‐pharmacological, the specific treatment or therapy administered (such as melatonin, CBTI‐BD, or bright light therapy (BLT)), the duration of the intervention, and the presence and nature of any control or comparison condition.

For each study, sleep‐related outcomes were documented based on the assessment instruments used, which included both subjective measures, such as the Pittsburgh Sleep Quality Index (PSQI) and the Insomnia Severity Index (ISI), as well as objective measures, including actigraphy. Reported sleep outcomes included changes in sleep quality, efficiency, duration, latency, total sleep time, and alignment of the circadian phase.

Clinical outcomes were also extracted, focusing on mood symptom measures, such as scores for depressive or manic symptoms, along with broader indicators of clinical progression, including relapse prevention, impulsivity, and functional status. Adverse events and tolerability issues reported during the study period were systematically noted, with attention to their nature, frequency, and severity.

### 2.5 Assessment of the Quality of Studies and Risk of Bias From the Review

The methodological quality of the included studies was independently evaluated by two reviewers using validated tools suitable for each study design. For randomized controlled trials (RCTs), the revised Cochrane risk‐of‐bias tool for randomized trials (RoB 2) was utilized [19]. This tool assesses five domains: Bias arising from the randomization process, deviations from intended interventions, missing outcome data, measurement of outcomes, and selection of the reported result. Each domain was classified as having low risk, some concerns, or high risk of bias, resulting in an overall risk‐of‐bias judgment for each study.

For non‐randomized studies, including cohort and cross‐sectional designs, the Risk Of Bias In Non‐randomized Studies of Interventions (ROBINS‐I) tool was utilized [20]. This instrument assesses potential biases across seven domains, such as confounding, participant selection, classification of interventions, deviations from intended interventions, missing data, outcome measurement, and selection of the reported result. Each domain and the overall study were rated as exhibiting low, moderate, serious, or critical risk of bias.

No quantitative synthesis was conducted because the included studies differed substantially in intervention type, comparator, outcome measures, and study design. Consequently, no pooled effect estimates were derived. Formal assessment of publication bias using funnel plot asymmetry or regression‐based tests was therefore not appropriate, as these methods require an adequate number of studies reporting a common effect measure and are unreliable when fewer than ten studies are available for a given outcome or when heterogeneity is substantial. Publication bias is instead considered narratively in the Discussion.

## 3 Results

### 3.1 Flow Chart of Included Studies

**FIGURE 1:**
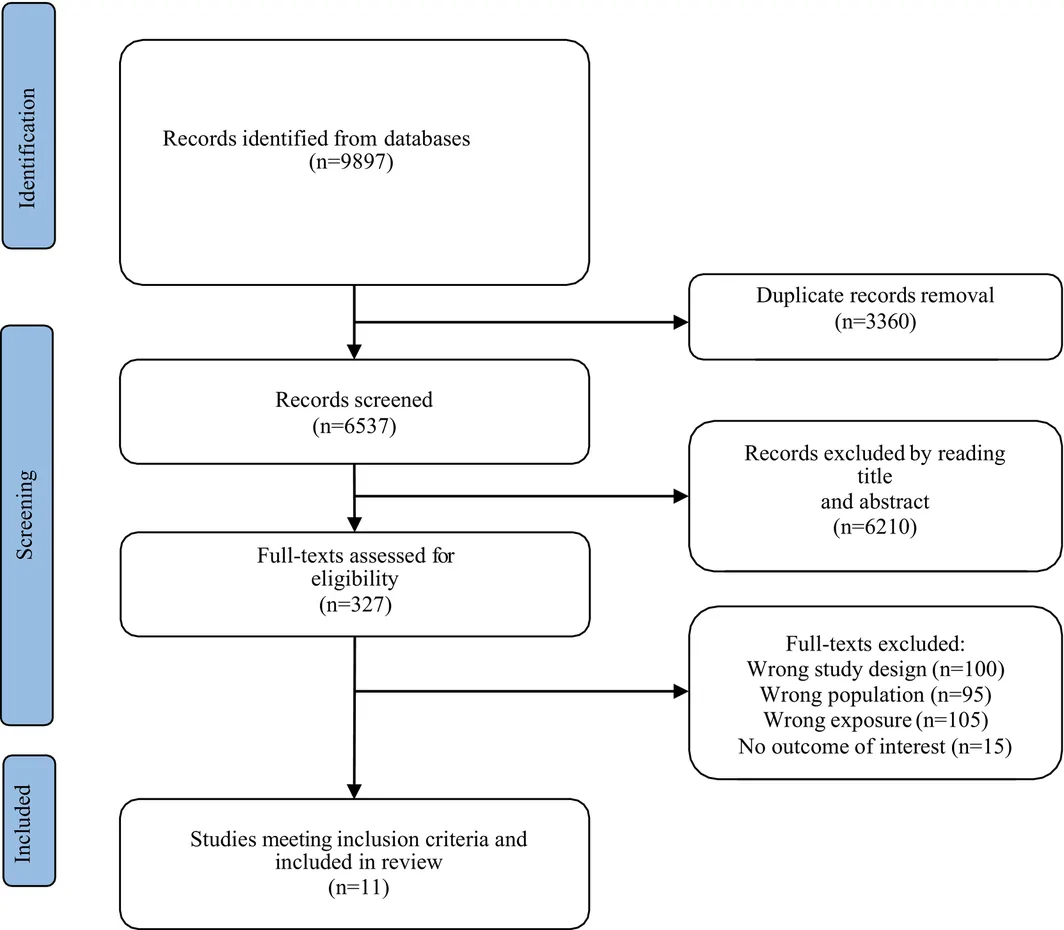
Flow‐chart describing the study selection process.

Search results are summarized in the PRISMA Flow Chart in Figure 1. Initially, 9897 studies were identified through the search strategy from the databases. After removing duplicates, 6537 remained. After title and abstract screening, 6210 did not meet our inclusion criteria and were therefore excluded. Finally, we included 11 studies in the systematic review (Table 1).

**TABLE 1:** Study characteristics of included studies.

Author/year	Country	Study design	Intervention type	Intervention description	N. participants	BD diagnosis	Clinical state	Duration of intervention
Cafaro et al., 2024	USA	RCT	Suvorexant	All participants who completed the randomized phase were offered a 3‐month open‐label treatment with suvorexant. Both subjective (sTST) and objective (oTST) total sleep time were measured.	36	BD Type I: 19 BD Type II: 15 BD NOS: 2	Euthymic/depressive	3 months
Cruz‐Sanabria et al., 2023	Italy	RCT	Melatonin	Patients meeting criteria for delayed sleep–wake phase disorder received a 3‐month treatment with 2 mg of exogenous melatonin (exo‐MEL), administered approximately 4 h before their actigraphically determined sleep onset time (SOT) at baseline.	19	BD Type I: 4 BD Type II: 15	Euthymic	3 months
Esaki et al., 2020	Japan	RCT	Blue‐blocking glasses	Patients were randomly assigned to wear blue‐blocking glasses and were instructed to use these from 20:00 h until bedtime.	43	BD Type I: 16 BD Type II: 27	Stabilized	2 weeks
Gharehbaghi et al., 2024	Iran	RCT	Benson relaxation technique	The intervention group practiced the Benson Relaxation Technique (BRT) twice daily (morning and evening) under the supervision of a trained psychiatric nurse. On the first day, participants received instruction on how to perform BRT; during the following sessions, the nurse remained bedside throughout the entire relaxation process.	60	BD Type I	Stabilized	3 weeks
Harvey et al., 2015	USA	RCT	CBTI‐BD OR psychoeducation	Alongside psychiatric care, interepisode bipolar disorder I participants with insomnia were randomly allocated to a bipolar disorder–specific modification of cognitive behavior therapy for insomnia (CBTI‐BD; *n* = 30) or psychoeducation (PE; *n* = 28) as a comparison condition. Outcomes were assessed at baseline, the end of 8 sessions of treatment, and 6 months later.	58	BD Type I	Interepisode	6 months
Kaplan et al., 2018	USA	RCT	RISE‐UP routine in CBTI	Testing the effectiveness of a morning routine called RISE‐UP to reduce sleep inertia in people with insomnia and bipolar disorder. Sleep and sleep inertia were monitored in the week prior to and following the intervention with daily sleep diaries, actigraphy, and ecological momentary assessment (EMA). Participants were randomized to a bipolar‐specific modification of CBT‐I (CBTI‐BD) with RISE‐UP (*N* = 20) or a psychoeducation (PE) comparison condition (*N* = 20).	40	BD Type I	Interepisode	15 months
Kupeli et al., 2018	Turkey	RCT	Bright light therapy	This study evaluates the efficacy and safety of BLT as an add‐on treatment for BD. Thirty‐two BD outpatients were randomly assigned to BLT (10,000 lx) or dim light (DL, < 500 lx). During a two‐week period, light was administered each morning for 30 min.	32	BD Type I: 17 BD Type II: 15	Depressive	2 weeks
Lee et al., 2015	USA	RCT	CBTI‐BD or psychoeducation	Examine the extent to which patients recall the contents of therapy from one session to the next and determine whether recall is associated with treatment outcome. Patients were randomly allocated to receive either CBTI‐BD or PE. Both treatment groups consisted of weekly one‐on‐one sessions lasting 50 min each for 8 consecutive weeks. At the beginning of each weekly session, patients freely recalled as many therapy points (i.e., distinct ideas, principles, and experiences) as they could from their previous session. After each session, therapists recorded a list of all therapy points delivered.	30	BD Type I	Interepisode	8 weeks
Sit et al., 2018	USA	RCT	BLT	Patients were randomly assigned to treatment with either 7,000‐lx bright white light or 50‐lx dim red placebo light. Symptoms were assessed weekly with the Structured Interview Guide for the Hamilton Depression Scale With Atypical Depression Supplement (SIGH‐ADS), the Mania Rating Scale, and the Pittsburgh Sleep Quality Index.	46	BD Type I: 31 BD Type II: 15	Depressive	6 weeks
Wang et al., 2025	China	Cross‐sectional	Systematic antidepressant treatments in the early stages	Patients were divided into AT group (systematic antidepressant treatment group) and NT group (no systematic antidepressant treatment group). Sleep quality and impulsivity were assessed using the Pittsburgh Sleep Quality Index and the Barratt Impulsivity Scale Questionnaire version 11, respectively.	124	NR	Euthymic	6 weeks at least
Zhou et al., 2018	China	RCT	BLT	Participants were randomized in one of two treatment conditions: BLT and control. All participants were required to complete several scale assessments at baseline and at the end of weeks 1 and 2. The primary outcome measures (clinical efficacy of BLT and the onset time of BLT) were assessed by the Hamilton Depression Rating Scale (HDRS) and the Quick Inventory of Depressive Symptomatology Self‐Report (QIDS‐SR16).	74	NR	Acute depressive	2 weeks

Abbreviations: BD = bipolar disorder; BLT = Bright Light Therapy; CBTI‐BD = Cognitive Behavioral Therapy for Insomnia adapted for Bipolar Disorder; DL = dim light; EMA = Ecological Momentary Assessment; exo‐MEL = exogenous melatonin; HDRS = Hamilton Depression Rating Scale; NOS = not otherwise specified; NR = not reported; oTST = objective total sleep time; PE = psychoeducation; RCT = randomized controlled trial; SIGH‐ADS = Structured Interview Guide for the Hamilton Depression Rating Scale—Atypical Depression Supplement; SOT = sleep onset time; sTST = subjective total sleep time.

### 3.2 Ratings of Study Quality and Risk of Bias

Risk of bias was assessed as described in Section 2.5; the full ratings are reported in Table 2.

**TABLE 2:** Risk of bias assessment of the included studies.

Author, year	Risk of bias tool	Overall risk of bias
Cafaro et al., 2024	Rob 2	Some concerns 🟡
Cruz‐Sanabria et al., 2023	Rob 2	Low 🟢
Esaki et al., 2020	Rob 2	Low 🟢
Gharehbaghi et al., 2024	Rob 2	Low 🟢
Harvey et al., 2015	Rob 2	Some concerns 🟡
Kaplan et al., 2018	Rob 2	Some concerns 🟡
Kupeli et al., 2018	Rob 2	Some concerns 🟡
Lee et al., 2015	Rob 2	Some concerns 🟡
Sit et al., 2018	Rob 2	Low 🟢
Wang et al., 2025	ROBINS‐I	Serious 🔴
Zhou et al., 2018	Rob 2	Low 🟢

Abbreviations: ROBINS‐I = Risk Of Bias In Non‐randomized Studies—of Interventions; ROB 2 = a revised tool to assess risk of bias in randomized trials.

Among the randomized controlled trials, most studies were rated as having either a low risk of bias or “some concerns,” primarily due to limitations in blinding, small sample sizes, or incomplete outcome reporting. Specifically, five RCTs [15, 16, 21, 22, 23] were rated as having low risk of bias. Another five RCTs [17, 24, 25, 26, 27] were judged to have “some concerns”, most often due to uncertainty in the randomization process or incomplete information on outcome measurement blinding.

For the single non‐randomized study [28], the ROBINS‐I tool identified a serious overall risk of bias. The primary concerns were related to the risk of confounding, potential selection bias, and limitations in classifying interventions. This study lacked clear control over confounding variables and employed an observational design without random assignment, which compromised the internal validity of the findings.

Overall, while the majority of RCTs were of moderate to high methodological quality, the non‐randomized study contributed additional contextual information but was interpreted with caution due to its greater susceptibility to bias. The results of the risk of bias assessments are presented in Table 2 and were considered in the interpretation of the review's findings.

### 3.3 Characteristics of the Included Studies

This review included 11 studies published between 2015 and May 2025, comprising 10 RCTs and one observational study (a cross‐sectional study). The studies were conducted across a range of geographic locations, including the USA, China, Iran, Japan, Italy, and Turkey. Sample sizes varied from 19 to 124 participants. Most studies recruited individuals diagnosed with bipolar disorder Type I or II, while a few included participants with unspecified subtypes.

The clinical status of participants at baseline varied: Some studies enrolled individuals during euthymic phases, others included those experiencing depressive or acute affective episodes. The interventions investigated encompassed both pharmacological and non‐pharmacological approaches. Pharmacological interventions included melatonin, suvorexant, and antidepressants. In contrast, non‐pharmacological strategies included CBTI‐BD, BLT, blue‐blocking glasses, the Benson relaxation technique, and psychoeducation.

The duration of interventions ranged from two weeks to nine months. Sleep outcomes were assessed using both subjective tools (e.g., PSQI, ISI, sleep diaries) and objective measures (e.g., actigraphy). Clinical outcomes included mood symptoms, relapse rates, aggression, impulsivity, and overall functioning. Tolerability and adverse events were reported across most studies, with minimal serious adverse effects observed.

### 3.4 Sleep Outcomes

Sleep outcomes were assessed in all 11 included studies, using a range of subjective and objective instruments. The most frequently used subjective measures were the PSQI, the ISI, sleep diaries, and the MEQ/rMEQ, the latter capturing self‐reported circadian preference. Actigraphy was the only objective instrument employed across the included studies.

Several studies reported significant improvements in sleep quality following psychological or behavioral interventions. CBTI‐BD was associated with increased sleep efficiency, reduced sleep latency, and improved sleep regularity in three trials [17, 25, 27]. The Benson relaxation technique also led to significant reductions in PSQI scores across multiple domains [16]. BLT, as investigated by Kupeli et al. [26], was associated with reductions in insomnia symptoms. In contrast, Sit et al. [22] observed non‐significant trends toward better sleep quality in participants exposed to 7,000 lx light. Blue‐blocking glasses, examined by Esaki et al. [21], induced a significant phase advance in chronotype, although no significant changes were observed in overall subjective sleep quality.

Among pharmacological studies, the use of exogenous melatonin was associated with earlier sleep onset, increased total sleep time, and higher sleep efficiency as measured by actigraphy and PSQI [15]. Suvorexant produced modest objective increases in total sleep time, though subjective sleep improvements were not statistically significant [24]. Wang et al. [28] evaluated systematic antidepressant treatment and reported poorer subjective sleep quality (PSQI) among treated patients, with no evidence of benefit on sleep parameters.

Overall, non‐pharmacological interventions produced the most consistent improvements in sleep quality across trials, whereas pharmacological interventions yielded more modest and heterogeneous effects, with objective gains not always matched by subjective improvement.

### 3.5 Clinical Outcomes

Clinical outcomes were variably reported across the included studies and primarily focused on mood symptoms (depressive and manic), aggression, impulsivity, and global functioning. All included interventions were sleep‐targeted by design. Beyond sleep parameters, most studies also assessed clinical outcomes, allowing the downstream effects of improved sleep on mood and related domains to be examined.

Clinical outcomes were derived from two distinct sources, which are specified for each study in Table 3. Mood symptoms were assessed predominantly through clinician‐rated instruments administered by trained raters, namely the IDS‐C, MADRS, HDRS, SIGH‐ADS, YMRS, and CGI; one trial additionally included a self‐reported depression measure (QIDS‐SR16). Self‐reported questionnaires were otherwise confined to secondary behavioral outcomes, specifically aggression, assessed with the Buss–Perry Aggression Questionnaire, and impulsivity, assessed with the Barratt Impulsiveness Scale (BIS‐11). This distinction is relevant to interpreting the findings since participant blinding was not feasible in the behavioral trials.

**TABLE 3:** Sleep intervention outcomes in included studies.

Author/year	Sleep measures	Sleep outcomes	Clinical outcomes	Outcome source	Adverse events
Cafaro et al., 2024	ACT	No significant improvements in subjective total sleep time (sTST); only a modest increase in objective total sleep time (oTST) with the Cole–Kripke algorithm during the RCT phase.	No significant differences observed in YMRS or MADRS scores between groups or over time.	Clinician‐rated	No adverse events or major intolerances were reported.
Cruz‐Sanabria et al., 2023	rMEQ/PSQI/ACT	Earlier sleep onset time (SOT), increased total sleep time (TST), higher sleep efficiency (SE), and significant increase in rMEQ scores after 3 months of exogenous melatonin treatment.	No affective symptoms were measured; all patients remained euthymic. Higher BMI was associated with smaller gains in sleep efficiency, and more hypomanic episodes were linked to smaller increases in total sleep time. Earlier melatonin intake correlated with greater improvement in sleep efficiency.	Clinician‐rated	No adverse events or tolerability issues reported.
Esaki et al., 2020	VAS/ISI/MEQ/ACT	No significant difference in sleep quality (VAS) between BB and placebo groups. MEQ scores shifted significantly toward morningness in the BB group. No significant differences in objective sleep parameters.	No significant differences in MADRS or YMRS scores. CGI scores showed marginally greater improvement in the BB group.	Clinician‐rated	No serious adverse events; mild discomfort or pain from glasses reported by 3 participants in the BB group and 4 in the placebo group. 1 mood worsening episode unrelated to intervention.
Gharehbaghi et al., 2024	PSQI	Significant improvements in sleep quality in the intervention group: Total PSQI score significantly reduced; improvements in subjective sleep quality, sleep latency, and sleep duration; 10% of participants achieved good sleep quality vs. 0% in control group.	BRT group showed significantly reduced total aggression scores compared to control. Reductions observed in physical aggression, verbal aggression, hostility, and anger.	Self‐report	No adverse events or tolerability issues reported.
Harvey et al., 2015	Sleep diaries, actigraphy, Sleep Disturbance and Sleep‐Related Impairment scales	Significant improvements in sleep efficiency, total sleep time, and variability in sleep/wake times in the CBT‐BD group vs. psychoeducation.	Significant reduction in depressive symptoms (IDS‐C) in the CBT‐BD group.	Clinician‐rated	No serious adverse events; some mild transient increases in sleepiness early in treatment
Kaplan et al., 2018	PSQI, sleep diaries, ISI	Improvements in sleep quality and insomnia severity in the CBTI‐BD compared to psychoeducation.	Reduction in MADRS in CBTI‐BD group; no significant differences in YMRS between groups.	Clinician‐rated	Mild, self‐limited sleep disturbances during early CBT sessions.
Kupeli et al., 2018	ISI, sleep diaries	Significant improvement in insomnia symptoms and sleep efficiency in the CBT‐I‐BD group compared to control.	Significant reductions in depression scores (BDI‐II) and anxiety.	Clinician‐rated	Some participants experienced initial sleep disruption, which resolved during the intervention.
Lee et al., 2015	PSQI, sleep diaries	Significant improvements in subjective sleep quality in the group receiving CBT‐I alongside pharmacotherapy compared to pharmacotherapy alone.	Improvement in depressive symptoms (HDRS) was greater in the combined treatment group.	Clinician‐rated	Mild and transient insomnia worsening in a few participants early in CBT‐I treatment.
Sit et al., 2018	PSQI	The active treatment group experienced better sleep quality compared with the inactive treatment group, but the differences were not significant.	The group treated with BLT experienced a significantly higher remission rate (68.2% compared with 22.2%; adjusted odds ratio = 12.6) at weeks 4–6 and significantly lower depression scores at the endpoint visit	Clinician‐rated	No serious adverse effects were reported. No mood polarity switches were observed.
Wang et al., 2025	PSQI	The average PSQI score of the AT (systematic antidepressant treatment) group (Mean = 8.27) was higher than the NT (no systematic antidepressant treatment) group (Mean = 6.13), and the difference was statistically significant (*p* < 0.001), showing poorer sleep quality.	Patients' sleep quality was positively correlated with impulsivity. Sleep quality mediated the relationship between antidepressant use and impulsivity, including both overall impulsivity and non‐planning impulsivity	Self‐report	Sleep quality and impulsivity during the euthymic period may be influenced by antidepressant use in the early stages.
Zhou et al., 2018	HDRS, YMRS, CGI, QIDS‐SR16	Not directly measured.	BLT, combined with first‐line drug medication, showed a greater ameliorative effect on bipolar depression than the control, with response rates of 78.19% vs. 43.33% respectively (*p* < 0.01) and faster onset time.	Clinician‐rated and self‐report	No serious adverse events were reported, neither increased rate of switching into hypomania.

Abbreviations: ACT = actigraphy; BB = blue‐blocking; BLT = bright light therapy; BMI = body mass index; BRT = Benson Relaxation Technique; CBTI‐BD = cognitive behavioral therapy for insomnia adapted for bipolar disorder; CGI = clinical global impression; DSM‐IV TR = Diagnostic and Statistical Manual of Mental Disorders, fourth edition, text‐revision; IDS‐C = Inventory of Depressive Symptomatology—clinician‐rated version; ISI = Insomnia Severity Index; IQR = interquartile range; MADRS = Montgomery and Asberg Depression Rating Scale; MDQ = Mood Disorder Questionnaire; MEQ = Morningness–Eveningness Questionnaire; oTST = objective total sleep time; PSQI = Pittsburgh Sleep Quality Index; QIDS = Quick Inventory of Depressive Symptomatology; rMEQ = reduced Morningness‐Eveningness Questionnaire; SE = sleep efficiency; SOT = sleep onset time; sTST = subjective total sleep time; VAS = Visual Analog Scale; YMRS = Young Mania Rating Scale.

Several non‐pharmacological interventions demonstrated beneficial effects on affective symptoms. CBTI‐BD, examined in three RCTs, was associated with significant reductions in depressive symptoms as measured by the Inventory of Depressive Symptomatology (IDS‐C) and the Montgomery–Åsberg Depression Rating Scale (MADRS) [17, 25, 27]. Improvements in sleep parameters often coincided with better emotional regulation and mood stability, though manic symptoms remained largely unaffected.

Relaxation‐based approaches also showed clinically relevant results. Gharehbaghi et al. [16] found that the Benson Relaxation Technique led to significant reductions in aggression, encompassing both physical and verbal aggression, as well as anger and hostility.

Light‐based interventions had mixed effects on mood. Sit et al. [22] reported a significantly higher remission rate from depression in the BLT group (68.2%) compared to placebo (22.2%), while Kupeli et al. [26] documented reductions in both depressive and anxiety symptoms. However, Esaki et al. [21] and Zhou et al. [23] reported more modest or non‐significant changes in depressive or manic symptomatology.

Pharmacological interventions produced variable results. Cruz‐Sanabria et al. [15] reported no change in affective symptoms, as all participants remained euthymic throughout the study. Cafaro et al. [24] similarly found no significant differences in depressive (MADRS) or manic (YMRS) symptoms. Wang et al. [28] reported that antidepressant use was associated with higher impulsivity, mediated by poorer sleep quality, raising concerns about unintended behavioral consequences.

These null findings for manic symptoms require cautious interpretation. Eight of the 11 included studies enrolled euthymic, stabilized, or interepisode participants, and no study enrolled participants in a manic or hypomanic state. As a result, baseline scores on manic symptom instruments such as the YMRS were close to the floor of the scale, leaving little room to detect further reduction.

### 3.6 Adverse Events and Tolerability

Adverse events and tolerability data were reported in most of the included studies, although the level of detail and systematic assessment varied considerably.

Non‐pharmacological interventions were generally well tolerated, with few reports of adverse effects. CBTI‐BD was associated with mild and transient increases in sleepiness or initial sleep disruption in the early phases of treatment [17, 25, 27]. The Benson Relaxation Technique and psychoeducational interventions were not associated with any reported adverse effects [16]. Among light‐based interventions, Sit et al. and Kupeli et al. reported no serious adverse events or mood polarity switches [22, 26]. Minor discomfort from blue‐blocking glasses was reported by a small number of participants in Esaki et al. [21], but no significant safety concerns emerged.

Pharmacological interventions presented a more variable tolerability profile. Suvorexant [24] and melatonin [15] were well tolerated, with no major adverse events or treatment‐related dropouts. No adverse events were systematically reported in the study by Wang et al. [28], though findings indicated a possible negative impact of antidepressant use on sleep and impulsivity.

Overall, non‐pharmacological interventions exhibited a more favorable safety profile, with minimal and self‐limited adverse effects. Pharmacological treatments demonstrated acceptable tolerability in controlled settings; however, their use in acute or comorbid conditions may warrant closer clinical monitoring due to potential behavioral risks.

## 4 Discussion

This systematic review synthesized evidence from randomized controlled trials and observational studies investigating pharmacological and non‐pharmacological interventions for sleep disturbances in individuals with BD. The findings suggest that non‐pharmacological interventions, particularly CBTI‐BD, are consistently associated with improvements in both subjective and objective sleep measures. These benefits often extend to mood symptoms, particularly depression. In contrast, pharmacological interventions produced more varied results, with melatonin and suvorexant showing moderate efficacy, while antidepressants raised safety concerns in certain clinical contexts.

Non‐pharmacological interventions have emerged as the most effective and safest options for addressing sleep disturbances in BD. CBTI‐BD was linked to significant improvements in sleep efficiency, latency, and total sleep time [25, 29], consistent with a systematic review of randomized trials of psychological and behavioral interventions targeting sleep and circadian rhythms in bipolar disorder [30], and aligning with prior evidence demonstrating the utility of CBT‐I in mood disorder populations [31, 32]. These effects are likely mediated through the stabilization of daily routines, correction of dysfunctional sleep beliefs, and restoration of circadian regularity—all of which are particularly relevant in BD, where social rhythm instability has been shown to trigger mood episodes [12, 33].

Light‐based therapies yielded heterogeneous results: While BLT improved depressive symptoms and insomnia in some trials [34, 35], others showed minimal effects, particularly on sleep‐related endpoints [36, 37]. This variability may reflect differences in treatment intensity, adherence, baseline circadian phenotype, or the timing of intervention, a factor known to influence efficacy in chronobiological treatments [38].

Pharmacological interventions produced less consistent results. Melatonin demonstrated positive effects on circadian alignment, sleep timing, and efficiency in euthymic patients with delayed sleep–wake phase disorder [39], aligning with existing literature on its utility in circadian rhythm disorders [40, 41]. Suvorexant, an orexin receptor antagonist, modestly increased total sleep time but failed to improve subjective sleep quality or mood symptoms significantly [42], a pattern partially observed in other studies using dual orexin antagonists in BD, where mood symptoms remained unchanged despite subjective sleep improvements [43]. Benzodiazepines and Z‐drugs, though commonly prescribed in clinical settings, were associated with limited therapeutic benefit and potential harm, including increased risk of suicidal behavior during acute episodes [44, 45], although no study eligible for the present review evaluated these agents. This finding is consistent with concerns that sedative‐hypnotics may worsen impulsivity or disinhibition in vulnerable individuals [46], and they may disrupt REM sleep architecture, which has been implicated in emotional regulation [47, 48]. The potential negative impact of antidepressants on sleep quality and impulsivity during euthymia [49, 50] highlights the need for careful monitoring, particularly since sleep disruption can itself mediate risk for affective switches [3, 5].

A structural feature of the available literature is that the clinical phase of enrolled participants covaried almost entirely with the intervention tested. The three CBTI‐BD trials [17, 25, 27] enrolled interepisode participants only; melatonin was evaluated only in euthymic participants with circadian misalignment [15]; bright light therapy was evaluated only during depressive episodes [22, 23, 26]; and blue‐blocking glasses [21] and the Benson relaxation technique [16] were evaluated only in stabilized participants. No intervention was tested across more than one clinical phase, and no study formally tested an interaction between clinical phase and treatment effect. The present review therefore cannot establish whether clinical phase moderates response to sleep‐targeted interventions: The apparent phase‐specificity of the results reflects the design of the existing evidence base rather than demonstrated moderation. This is itself a substantive gap, since phase‐specific treatment selection is precisely what clinicians require. Evidence from outside this review suggests that clinical state and chronotype may modulate treatment response [11, 51], and evening chronotype has been associated with greater mood instability and suicidality in BD [52, 53], but these remain hypotheses to be tested rather than conclusions supported by the trials reviewed here.

Clinical phase also constrains what can be measured: Interpreting manic symptom outcomes is complicated by the limited validity of manic symptom scales administered outside acute episodes, where items indexing elevated mood, energy, or sociability may capture normal‐range affective variation rather than residual pathology.

This review extends prior research by integrating recent trials and explicitly contrasting pharmacological and behavioral strategies across mood phases and circadian profiles. While earlier reviews emphasized the high prevalence of sleep disruption in BD [3, 5], fewer explored the nuanced impact of treatment modality or timing. Moreover, by incorporating both subjective and objective sleep metrics, this review highlights the value of actigraphy and sleep diaries as complementary tools, reinforcing the need for a multidimensional assessment of sleep in psychiatric populations [54].

The clinical relevance of these findings is substantial. Sleep interventions should not be seen as ancillary to mood stabilization but rather as integral components of relapse prevention strategies. Routine screening for insomnia and circadian misalignment should be implemented, particularly given evidence that sleep disruption often precedes mood episodes [5, 9]. CBTI‐BD and other non‐pharmacological strategies should be prioritized when feasible, and pharmacological options should be selected based on the phase of illness, chronotype, and risk profile. The risks associated with benzodiazepines, especially during mood episodes, necessitate greater caution and consideration of safer alternatives.

Despite encouraging results, several methodological limitations undermine the strength of the conclusions. The number of studies remains limited, and sample sizes were often small. Heterogeneity in outcome measures, participant characteristics, and follow‐up durations complicates comparisons across trials. Several constraints also limit what can be inferred from these data. Because each intervention was evaluated in a single clinical phase, the effects of intervention type cannot be separated from those of clinical state, and no conclusions regarding moderation by clinical phase are possible. Few studies tracked relapse rates over time, and although manic symptoms were assessed in several trials, they were assessed almost exclusively in euthymic or interepisode samples in which floor effects preclude the detection of change. This review is therefore not positioned to draw conclusions regarding the effect of sleep‐focused interventions on manic symptomatology; trials enrolling participants during, or at elevated risk of, manic episodes would be required. Floor effects do not, however, constrain the detection of symptom emergence, so the absence of treatment‐emergent affective elevation across the included studies remains informative with respect to tolerability.

Moreover, no included study used a physiological marker of circadian phase, such as dim‐light melatonin onset; conclusions regarding circadian realignment therefore rely on self‐reported preference and on actigraphy‐derived rest–activity patterns.

Publication bias could not be formally assessed and cannot be ruled out. The included trials were predominantly small, and small trials with positive findings are more likely to be published than those with null findings. Our search was further restricted to peer‐reviewed articles published in English between January 2015 and May 2025, and neither gray literature nor trial registries were searched. The overall direction of effect reported here, particularly for behavioral interventions, may therefore be more favorable than the underlying evidence base. Finally, clinical outcomes derived from different sources: Findings based on clinician‐rated instruments are less susceptible to participant expectancy effects, although they remain vulnerable to rater unblinding, whereas self‐reported outcomes in unblinded behavioral trials should be interpreted more cautiously.

Future research should focus on large‐scale, phase‐specific randomized trials that stratify participants by chronotype and mood state. Interventions should include both objective (e.g., actigraphy, dim light melatonin onset) and subjective sleep measures, assessing long‐term outcomes such as relapse, functional recovery, and suicidality. Integrating wearable technology and ecological momentary assessment may facilitate personalized sleep monitoring and adaptive interventions. Combining behavioral and pharmacological strategies, such as CBTI‐BD with melatonin, could also enhance treatment outcomes. Finally, mechanistic studies examining sleep‐dependent emotional regulation, REM disruption, and circadian gene polymorphisms (e.g., CLOCK, PER3) may provide valuable insights into treatment personalization [55, 56].

Sleep disturbances in BD are pervasive, clinically meaningful, and modifiable. The evidence reviewed here indicates that behavioral interventions, particularly CBTI‐BD, yield the most consistent improvements in sleep and the most favorable tolerability profile, with reductions in depressive symptoms. Pharmacological strategies may be helpful in selected cases but require careful consideration of timing, tolerability, and underlying chronobiological factors. Whether treatment should be tailored to clinical phase and chronotype remains an open question: Each intervention in this review was evaluated in a single clinical phase, so the available trials cannot establish whether these factors moderate treatment response. Designing trials capable of detecting such moderation is a priority for future research and a precondition for genuinely individualized treatment recommendations in bipolar disorder.

## Author Contributions

V.B. conceptualized and designed the study, led the conduct of the systematic search and data analysis, and drafted the manuscript. F.P. and F.I. contributed to the systematic search. N.V. contributed to the systematic search and overall study design. D.D.R., G.P., and L.P. contributed to the overall study design. M.M. contributed to the overall study design. All authors provided critical revisions of the manuscript for important intellectual content. All authors read and approved the final manuscript.

## Conflicts of Interest

The authors declare no conflicts of interest.

## Data Availability

Data sharing not applicable to this article as no datasets were generated or analysed during the current study.
